# Chronic Inflammation and Coronary Microvascular Dysfunction

**DOI:** 10.31083/RCM51873

**Published:** 2026-09-18

**Authors:** Huijing Yao, Jiangwei Cheng, Luqun Yang, Ruiying Wang, Yuanyuan Lin

**Affiliations:** ^1^Department of Cardiovascular Medicine, Third Hospital of Shanxi Medical University, Shanxi Bethune Hospital, Shanxi Academy of Medical Sciences, Tongji Shanxi Hospital, 030032 Taiyuan, Shanxi, China

**Keywords:** coronary microvascular dysfunction, chronic inflammation, microvascular homeostasis, therapeutic interventions

## Abstract

Coronary microvascular dysfunction (CMD) represents a pivotal pathological hallmark across diverse cardiovascular pathologies, the progression of which is tightly coupled to systemic chronic inflammation. Clinically, CMD presents as ischemia with non-obstructive coronary arteries (INOCAs) and myocardial infarction with non-obstructive coronary arteries (MINOCAs), and contributes substantially to heart failure with preserved ejection fraction (HFpEF). Moving beyond conventional risk-factor stratification, this review dissects the mechanistic pathways through which individual comorbidities compromise coronary microcirculatory integrity. In hypertension, increased mechanical wall stress activates proinflammatory Nuclear factor κB (NF-κB)–mitogen-activated protein kinases (MAPK) signaling cascades. In contrast, in diabetes, chronic hyperglycemia drives microvascular injury through the reactive oxygen species (ROS)–advanced glycation end-products (AGEs) axis and Protein Kinase C (PKC)-dependent pathways. In obesity, adipokine dysregulation and gut-derived metabolites, notably trimethylamine N-oxide (TMAO), promote NLR family pyrin domain containing 3 (NLRP3) inflammasome activation, a central mechanism in obesity-related CMD. In the setting of heart failure (HF) and cardiomyopathies, damage-associated molecular pattern (DAMP)–Toll-like receptor (TLR) interactions and TGF-β/Smad signaling promote capillary rarefaction and interstitial fibrosis, thereby linking systemic inflammation to structural microvascular remodeling. Furthermore, both autoimmune-mediated endotheliitis and virus-induced cytokine surges reduce nitric oxide (NO) bioavailability and impair coronary flow reserve (CFR). Thus, by integrating emerging therapeutic strategies, including interleukin-1β (IL-1β) antagonists, SGLT2 inhibitors, and selected traditional medicines, we highlight approaches aimed at restoring microvascular homeostasis. Finally, we propose a personalized framework that leverages multi-omics profiling and artificial intelligence (AI) to modulate the inflammatory milieu, positioning inflammation-targeted interventions as a fundamental strategy for CMD prevention and management.

## 1. Introduction

Recent shifts in clinical paradigms have increasingly positioned CMD not merely as a bystander but as a fundamental and independent determinant of myocardial ischemia that extends far beyond the traditional focus on obstructive coronary artery disease (CAD) [1,2]. CMD is characterized by structural remodeling and functional impairment of intramyocardial vessels, typically less than 500 µm in diameter, resulting in a critical mismatch between coronary blood flow (CBF) and myocardial oxygen demand [3]. Where epicardial CAD is defined by focal dynamics of atherosclerotic plaques, the hallmark of CMD lies in the diffuse, maladaptive architectural shifts and impaired vasoreactivity of the microcirculatory bed. Although endothelial and smooth muscle cell dysfunctions represent shared pathogenic pathways across the vascular tree, CMD manifests earlier and with greater sensitivity as a biomarker of cardiovascular vulnerability, correlating robustly with adverse major cardiovascular events (MACE) [4]. Broad-spectrum and targeted anti-inflammatory therapies have demonstrated efficacy in reducing cardiovascular risk among patients with obstructive CAD [5,6]; however, whether these benefits extend to microvasculature-mediated ischemia remains under active investigation. Emerging evidence identifies the systemic inflammatory milieu as a primary driver, rather than a secondary consequence, of CMD initiation and progression [7,8]. Elevated circulating biomarkers, including high-sensitivity C-reactive protein (hs-CRP) and various proinflammatory interleukins, correlate directly with the severity of microvascular impairment and are far from incidental findings [9]. Unlike acute inflammatory responses, the chronic inflammation observed in this context manifests as a protracted, insidious immunometabolic state characterized by persistent activation of both innate and adaptive immune pathways [10]. This systemic milieu, precipitated by a spectrum of triggers ranging from metabolic derangements to autoimmune stressors, orchestrates a deleterious cascade of mediators that inflict cumulative damage on the coronary microvasculature [11]. The specific roles of these mediators and their molecular targets in CMD are detailed in Table 1.

**Table 1.** **Classification and functions of common inflammatory mediators and cytokines**.

Mediator	Classification	Principal sources	Impact on coronary microvasculature	Key signaling axes
TNF-α	Cytokine	Macrophages, Adipocytes	Induces endothelial apoptosis; promotes microvascular rarefaction.	NF-κB, MAPK
IL-1β	Cytokine	NLRP3 Inflammasome	Triggers “cytokine priming”; impairs NO bioavailability.	NLRP3/NF-κB
TGF-β	Growth Factor	Fibroblasts, Macrophages	Promotes EndMT; exacerbates perivascular fibrosis.	Smad2/3, MAPK
TMAO	Metabolite	Gut Microbiota	Activates NLRP3; impairs endothelial glycocalyx integrity.	NLRP3/MAPK
CRP	Acute-phase Protein	Liver (IL-6 induced)	Direct pro-inflammatory insult; impairs vasodilator recruitment.	IL-6/JAK/STAT
Adiponectin	Adipokine	Adipocytes	Vasoprotective; enhances NO production (Downregulated in CMD).	AMPK/PI3K/eNOS
HMGB1	DAMPs	Necrotic cells	Acts as a “danger signal”; initiates microvascular endotheliitis.	TLR4/RAGE

TNF-α, Tumor necrosis factor-α; IL, Interleukin; TGF-β, Transforming growth factor-β; TMAO, trimethylamine N-oxide; CRP, C-reactive protein; HMGB1, High mobility group box 1; NLRP3, NLR family pyrin domain containing 3; DAMPs, damage-associated molecular patterns; NF-κB, Nuclear factor κB; MAPK, mitogen-activated protein kinases; JAK, Janus kinase; STAT, Signal transducer and activator of transcription; AMPK, AMP-activated protein kinase; PI3K, Phosphoinositide 3-kinase; eNOS, endothelial nitric oxide synthase; TLR4, Toll-like receptor 4; RAGE, Receptor for advanced glycation end products.

This review aims to bridge the existing mechanistic gap by synthesizing how systemic inflammatory drivers originating from comorbidities, including hypertension, diabetes, and autoimmune disorders (Fig. 1), converge to disrupt coronary microvascular homeostasis. We further associate the clinical phenotypes of INOCA, MINOCA, and HFpEF with these underlying mechanisms, while critically evaluating the therapeutic potential of anti-inflammatory strategies in restoring microcirculatory function.

**Fig. 1.**
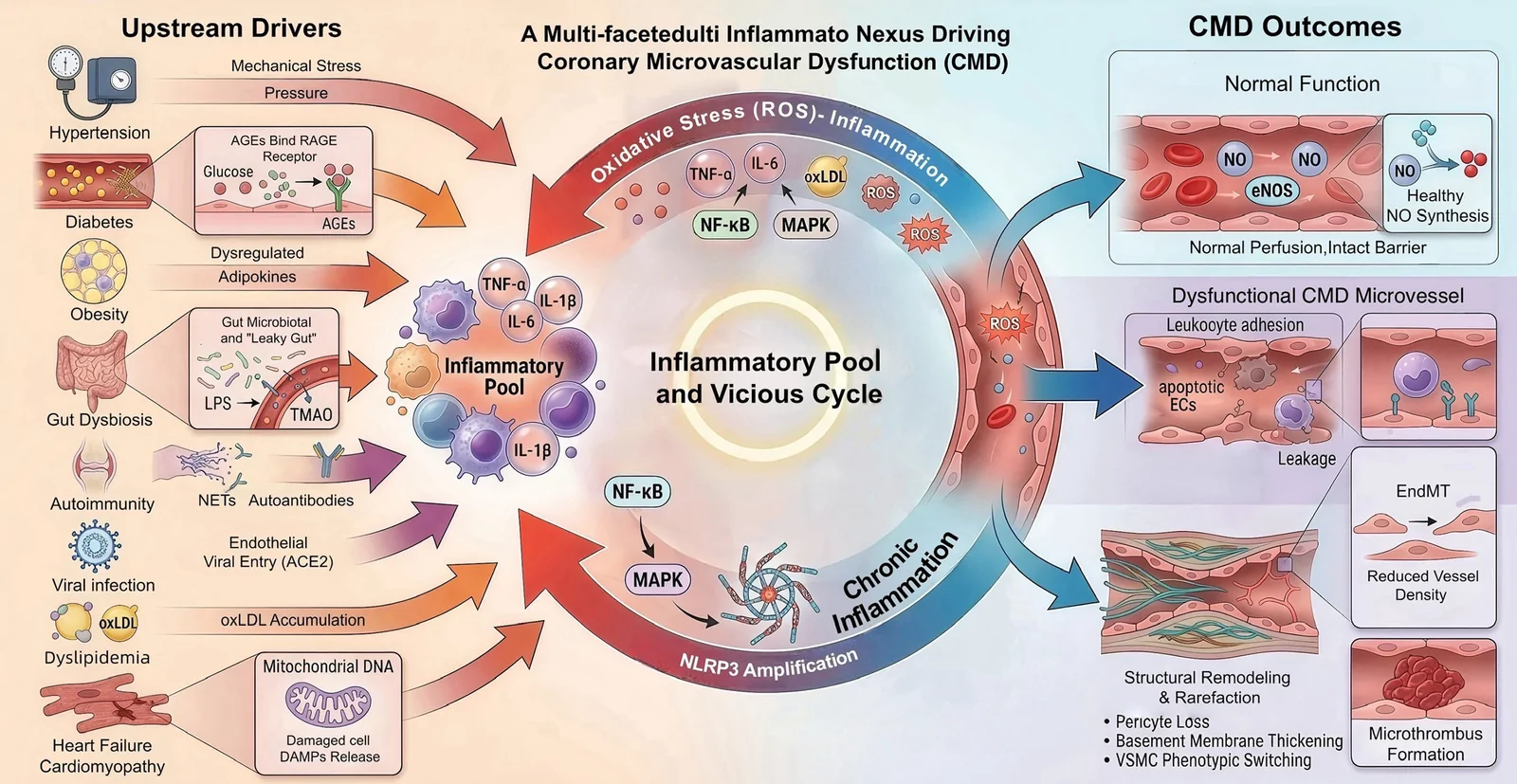
**Pathophysiological drivers and inflammatory mechanisms of CMD**. Systemic stimuli, including mechanical stress, metabolic derangements, and gut dysbiosis, converge to prime a systemic inflammatory pool. This triggers a self-perpetuating cycle of oxidative stress and chronic inflammation, mediated by NF-κB/MAPK signaling and NLRP3 inflammasome activation. These processes drive the transition from healthy NO-dependent microvascular homeostasis to CMD, characterized by endothelial dysfunction (leukocyte adhesion, apoptosis), structural remodeling (pericyte loss, basement membrane thickening), and vascular rarefaction (EndMT and microthrombosis).

## 2. The Role of Inflammation in Comorbidity-Driven CMD

### 2.1 Hypertension: From Mechanical Stress to Microvascular Remodeling

Hypertension is a primary driver of CMD, in which chronic hemodynamic loading establishes a substrate for microvascular-mediated ischemia independent of obstructive CAD [11,12]. Although hypertensive insult affects the entire vascular tree, the mechanisms underlying microcirculatory failure differ fundamentally from those governing epicardial atherosclerosis. Within the microcirculation, pathological wall stress is transduced into biochemical inflammatory cascades. Crucially, endothelial cells (ECs), acting as mechanosensors, undergo maladaptive phenotypic shifts in response to hypertensive stress [13], accompanied by dedifferentiation of contractile vascular smooth muscle cells (VSMCs) toward a synthetic, pro-inflammatory state. This transition involves upregulation of mitogen-activated protein kinases (MAPK) and NF-κB, which subsequently induce matrix metalloproteinases (MMPs) [14,15]. Among these, NF-κB serves as the central signaling hub, integrating mechanical stress into sustained inflammation [16]; renin-angiotensin-aldosterone system (RAAS) inhibitors confer microvascular protection partly by attenuating this cascade [17]. Hypertension is an independent risk factor for INOCA [18]. Clinically, hypertensive patients with CMD often present with INOCA, particularly in postmenopausal women; the acute presentation of this condition can be MINOCA. Moreover, chronic microvascular rarefaction and diastolic dysfunction further predispose these patients to HFpEF [18,19]. Mechanistically, these signaling hubs recruit and activate T-cells and macrophages, fostering a localized inflammatory milieu that precipitates vascular tissue damage [20,21,22]. Simultaneously, this environment exacerbates oxidative stress, which synergizes with NF-κB activation to downregulate tight junction proteins such as claudin-5, thereby compromising endothelial barrier integrity and increasing microvascular permeability [23,24]. Specifically, the aberrant secretion of MMP-2 and MMP-9 catalyzes extracellular matrix degradation, resulting in structural remodeling and microvascular rarefaction, a reduction in capillary density that constitutes a hallmark of hypertensive CMD [25]. These interconnected processes establish a self-perpetuating vicious cycle: elevated microvascular resistance further attenuates coronary flow reserve (CFR), thereby driving concurrent progression of systemic hypertension and microcirculatory failure [26].

### 2.2 Diabetes: Metabolic Inflammation and the Microvascular Triad

Diabetes mellitus induces persistent, low-grade systemic inflammation that poses a profound threat to microvascular stability. Although hyperglycemia accelerates epicardial atherosclerosis through foam cell accumulation, the diabetic coronary microcirculation is distinguished by a characteristic triadic lesion: capillary basement membrane thickening, pericyte loss, and impaired endothelium-dependent vasodilation [27,28]. Hyperglycemia-driven mitochondrial oxidative stress establishes a pro-inflammatory “metabolic memory” via epigenetic modifications, sustaining the ROS-AGEs axis and receptor for advanced glycation end products (RAGE) signaling even after glycemic normalization [29,30,31,32,33,34,35].

Chronic inflammation constitutes the cornerstone of this diabetic microcirculopathy [7]. The accumulation of Advanced Glycation End Products (AGEs) enhances leukocyte-endothelial adhesion and upregulates adhesion molecules, such as intercellular adhesion molecule 1 (ICAM-1) and vascular cell adhesion molecule 1 (VCAM-1), promoting inflammatory infiltration into the perivascular space. Concurrently, the sustained activation of Protein Kinase C (PKC) and NF-κB triggers endothelial-to-mesenchymal transition (EndMT), shifting ECs toward a pro-fibrotic phenotype that impairs barrier function [27,28]. Conversely, endogenous anti-inflammatory molecules, including Interleukin-37 (IL-37) and IL-10, serve as vital counter-regulatory mechanisms; IL-10, in particular, exerts cardioprotective effects by modulating NO bioavailability and mitigating the oxidative damage secondary to chronic inflammatory processes [36,37,38]. Collectively, these metabolic-inflammatory perturbations culminate in a profound reduction of the microvascular exchange surface and an impaired hyperemic response, driving the clinical manifestations of diabetic CMD. Clinically, diabetic CMD underlies INOCA and predicts HFpEF progression [39,40]. SGLT2 inhibitors (SGLT2i) suppress the ROS-AGEs-NF-κB axis, restore NO bioavailability, and reduce HFpEF hospitalizations [41,42]. Notably, diabetes is an independent risk factor for MINOCA, as hyperglycemia-induced microvascular spasm and thrombosis can precipitate myocardial injury in the absence of epicardial obstruction [43].

### 2.3 Obesity: Adiposopathy and the Pro-Inflammatory Adipo-Vascular Axis

Obesity is defined by a pathological accumulation of adipose tissue that poses significant systemic health risks [44]. Beyond simple energy sequestration, adipose tissue serves as a dynamic endocrine and immunomodulatory organ; white adipose tissue (WAT), in particular, comprises a complex cellular network of adipocytes, fibroblasts, and immune cells [45]. Adipose tissue maintains systemic metabolic homeostasis through the secretion of bioactive adipokines, including Plasminogen Activator Inhibitor Type-1 (PAI-1), Tumor necrosis factor-α (TNF-α), Interleukin‑6 (IL-6), leptin, and adiponectin [46,47,48,49,50,51].

Obesity promotes a chronic, low-grade inflammatory state, termed “adiposopathy”, characterized by adipocyte hypertrophy, localized hypoxia, and oxidative stress. A hallmark of this endocrine derangement is a secretory shift: pro-inflammatory mediators are overproduced while vasoprotective adiponectin is markedly downregulated [52]. Mechanistically, the activation of the NLRP3 inflammasome within expanded adipose tissue drives systemic release of IL-1β and IL-18. These cytokines directly insult the coronary endothelium, increasing vascular permeability and inducing a pro-thrombotic microenvironment through elevated PAI-1 expression, creating a microenvironment prone to thrombosis. This represents a key node linking visceral adipose accumulation to microvascular injury [53]. Weight loss induced by glucagon-like peptide-1 receptor agonists (GLP-1 RAs) has been shown to suppress NLRP3 inflammasome activation in adipose tissue while concurrently improving myocardial perfusion reserve index (MPRI) on cardiac magnetic resonance imaging [54,55]. Moreover, systemic elevation of chemokines facilitates the recruitment of macrophages to the perivascular space, further inducing pathological microvascular remodeling [56,57].

Critically, obesity-induced oxidative stress exhibits a bidirectional synergy with adipokine dysregulation [58]. Hypoxia-activated nicotinamide adenine dinucleotide phosphate (NADPH) oxidase in hypertrophic adipocytes generates excessive reactive oxygen species (ROS), causing direct oxidative damage to endothelial membrane phospholipids and genomic deoxyribonucleic acid (DNA), thereby accelerating apoptosis [59]. ROS simultaneously impair the phosphorylation of endothelial nitric oxide synthase (eNOS), severely reducing NO bioavailability. This deficit not only compromises microvascular vasodilatory capacity but also drives VSMC phenotypic switching toward a proliferative state. The resulting medial hypertrophy and luminal narrowing substantially reduce myocardial blood flow reserve (MBFR) [60,61]. This self-perpetuating cycle of oxidative stress and inflammatory signaling disrupts the structural integrity of the coronary microcirculation, serving as a cornerstone of obesity-related CMD. Emerging evidence further implicates obesity-driven CMD as a pivotal contributor to myocardial fibrosis, increased cardiomyocyte stiffness, and the development and progression of HFpEF [62].

### 2.4 HF: DAMPs, Neurohumoral Activation, and Myocardial Stiffness

In HF, the collapse of hemodynamic compensation precipitates myocardial injury and systemic hypoperfusion, igniting a systemic immunometabolic response [63,64]. This inflammatory milieu critically governs both myocardial remodeling and the progression of CMD [65,66]. Within the failing myocardium, cardiomyocyte necrosis, interstitial fibrosis, and extracellular matrix degradation collectively release damage-associated molecular patterns (DAMPs), including HMGB1, heat shock proteins, and mitochondrial DNA, into the microenvironment [67]. These endogenous ligands engage pattern-recognition receptors (PRRs), notably TLR4, on leukocytes and cardiomyocytes [68,69,70,71,72]. This interaction triggers downstream NF-κB signaling, culminating in a cytokine storm of TNF-α, IL-1β, and IL-6 that compromises endothelial continuity and microvascular integrity [67,73,74,75]. Parallel to these immune responses, HF is characterized by persistent neurohumoral activation, notably via the RAAS. Angiotensin II (Ang II) exacerbates this inflammatory state by stimulating IL-6 secretion and synergizing with pro-inflammatory adipokines, particularly in patients with comorbid obesity or hypertension [75,76]. A central mechanistic hallmark of HF-related CMD is uncoupling of the NO-cGMP-PKG signaling axis. Inflammation-driven ROS elevate peroxynitrite (ONOO⁻) levels, suppressing cGMP production by soluble guanylate cyclase (sGC). The consequent loss of PKG activity removes the constitutive brake on cardiomyocyte hypertrophy and impairs titin-mediated relaxation, thereby increasing diastolic stiffness [77,78,79,80,81,82]. In the PROMIS-HFpEF study, 151 of 202 HFpEF patients exhibited CMD (CFR <2.5), with impaired NO-cGMP-PKG signaling identified as the core mechanism [40]. Existing studies have demonstrated that sGC stimulators, such as vericiguat and riociguat, can ameliorate HFpEF by targeting the NO-sGC-cGMP pathway [83]. In parallel, upregulated VCAM-1 and E-selectin promote transendothelial migration of leukocytes; infiltrating cells release TGF-β, driving myofibroblast differentiation and interstitial collagen deposition [84]. Furthermore, inflammatory mediators also induce VE-cadherin phosphorylation, destabilizing inter-endothelial junctions and leading to microvascular leakage and myocardial edema [85]. Together, these structural and functional perturbations establish a vicious cycle in which microvascular failure and myocardial dysfunction mutually reinforce the progression of CMD.

### 2.5 Cardiomyopathy: Genetic Priming and Sterile Inflammation

Diverse cardiomyopathies, including dilated and hypertrophic phenotypes, are characterized by a distinctive sterile inflammatory signature that drives microvascular remodeling. Hypertrophic cardiomyopathy (HCM), a prototypical monogenic disorder caused by sarcomeric mutations, exemplifies the mechanoinflammatory nexus linking genetic defects to CMD and sudden cardiac death (SCD) [86]. Translational evidence from the MYH7 R453C porcine model indicates that genetic mutations trigger the aberrant activation of TGF-β/Smad2/3 and PI3K-Akt signaling pathways, simultaneously upregulating ROS production via NADPH oxidase 4 (Nox4). This establishes a Nox4/ROS/NF-κB axis that serves as a central hub for maladaptive cardiac remodeling and impaired angiogenesis. Consequently, the synergistic activation of TGF-β and Nox4 pathways promotes endothelial senescence and mitochondrial dysfunction, culminating in endothelial apoptosis and progressive myocardial fibrosis [87,88,89,90,91].

Emerging evidence further implicates NETosis, a specialized form of neutrophil-mediated thrombo-inflammation, as a novel driver of microvascular impairment in HCM. Neutrophil extracellular traps (NETs), composed of cell-free DNA (cfDNA) complexed with cytotoxic histones, directly damage coronary endothelial membranes through proteolytic and oxidative mechanisms. These web-like structures activate the complement cascade and promote a pro-thrombotic microenvironment, severely compromising microcirculatory patency within the hypertrophied myocardium [92].

Central to this sterile immune response is the NLRP3 inflammasome, which functions as a high-sensitivity sensor of cellular stress that amplifies local inflammation by cleaving pro-interleukins into bioactive IL-1β and IL-18, occasionally triggering pyroptosis [93]. Clinical studies indicate that systemic NF-κB activity in HCM patients provides a robust priming signal for NLRP3 assembly, with elevated inflammatory indices such as high-sensitivity CRP serving as independent predictors of adverse long-term prognosis [94]. Sustained NLRP3 activation establishes a self-amplifying loop of IL-1β release that upregulates endothelial tissue factor (TF) expression. The resulting microthrombosis and capillary rarefaction reduce the microvascular exchange surface, thereby perpetuating CMD in the setting of genetic cardiomyopathy [95,96]. This process is closely associated with adverse long-term left ventricular remodeling and progression to HF [97].

### 2.6 Systemic Autoimmune Disorders: Subclinical Drivers of CMD

Systemic autoimmune disorders, including rheumatoid arthritis (RA), systemic lupus erythematosus (SLE), and psoriasis, arise from the breakdown of immune tolerance, establishing an insidious and persistent pro-inflammatory milieu. These conditions frequently manifest as “clinically silent” or subclinical CMD, which constitutes a potent independent predictor of future adverse cardiovascular events [98,99]. Microvascular impairment is driven by disease-specific cytokine signatures: a significant reduction in CFR can be observed in patients with inflammatory diseases, even in early stages and in the absence of obstructive CAD [100].

#### 2.6.1 RA: The MCP-1/MCPIP1 Axis

In RA, systemic inflammation characterized by elevated MCP-1 is tightly linked to impaired endothelium-dependent relaxation. Joint-localized inflammation triggers the expression of MCP-1-induced protein 1 (MCPIP1), which functions as a convergent pathological hub [101]. Specifically, MCPIP1 orchestrates NF-κB transactivation and amplifies systemic oxidative stress, thereby attenuating endothelial Akt activity and compromising eNOS-derived NO bioavailability. This axis is further marked by the upregulation of endothelin-1 (ET-1), predisposing the coronary microvasculature to vasospasm and structural remodeling [101,102,103,104]. Notably, statin therapy has been shown to downregulate MCPIP1, highlighting its therapeutic potential for reversing microvascular dysfunction in RA [105,106,107].

#### 2.6.2 SLE: IFN-α and Thrombo-Inflammation

The “interferon-alpha (IFN-α) signature” serves as a primary vascular toxin in SLE. Elevated IFN-α levels suppress NO synthesis by inducing miR-155, which in turn accelerates the degradation of eNOS regulatory genes [108,109]. Beyond direct endothelial injury, IFN-α primes neutrophil NET release, driving a specialized thrombo-inflammatory response that impairs microvascular patency [110]. Concurrently, IFN-α promotes the secretion of mature IL-18 via inflammasome activation in circulating angiogenic cells, further impairing the intrinsic capacity for vascular repair [111]. Anifrolumab, a type I interferon receptor antagonist, blocks the action of IFN-α in clinical practice, shows a favorable benefit-risk profile in patients with moderate-to-severe SLE on standard therapy, and ameliorates CMD attributable to multiple mechanisms to some extent [112]. The deposition of anti-dsDNA and antiphospholipid antibodies (APL) exacerbates this insult by triggering complement cascades, culminating in increased endothelial permeability and localized microthrombosis [111,113,114,115]. Notably, MINOCA is common in patients with antiphospholipid antibody syndrome (APLS) and is more frequent than in the non-APLS population [116].

#### 2.6.3 Psoriasis: The IL-17A-Driven Inflammatory Bed

The excess cardiovascular risk observed in psoriasis is largely attributable to CMD, which correlates independently with disease severity (Psoriasis Area and Severity Index (PASI) score) and duration [117].

Mechanistically, systemic IL-17A drives microvascular failure by activating TAK1/IKK and MAPK cascades upon binding to IL-17RA [118].

This molecular activation upregulates endothelial VCAM-1 and E-selectin, promoting robust leukocyte adhesion and transendothelial migration. Furthermore, IL-17A synergizes with TNF-α to suppress eNOS activity, impairing endothelium-dependent vasodilation and significantly reducing CFR [119,120].

### 2.7 Gut Microbiota: The Gut-Heart Axis in CMD

Emerging evidence positions the gut microbiota as a distal yet potent regulator of coronary microvascular health. Gut dysbiosis, characterized by a reduction in microbial diversity and the proliferation of pathobionts, facilitates the systemic translocation of lipopolysaccharide (LPS). This endotoxin acts as a primary ligand for Toll-like receptor 4 (TLR4), triggering NF-κB–mediated pro-inflammatory signaling that impairs both microvascular tone and endothelial barrier integrity. Central to the “gut-heart axis” is trimethylamine N-oxide (TMAO), a microbial metabolite derived from dietary choline and L-carnitine. TMAO has been shown to exacerbate CMD by promoting the assembly of the NLRP3 inflammasome and accelerating the recruitment of pro-inflammatory monocytes to the microvascular wall. These microbial-derived insults synergize with conventional risk factors to accelerate endothelial senescence and microvascular rarefaction, highlighting the gut microbiome as a potential therapeutic target for restoring microcirculatory homeostasis [37,38]. Clinically, statins and angiotensin-converting enzyme inhibitors (ACEI)/angiotensin II receptor blockers (ARBs) improve CMD partly by inhibiting NLRP3 inflammasome activation, thereby alleviating symptoms of INOCA [121]. Meanwhile, the development of TMAO production inhibitors may become an important research direction for improving INOCA symptoms in the future.

### 2.8 Viral Infection: Endotheliitis and the Cytokine Storm

Viral infections, particularly COVID-19, have provided critical insights into acute-on-chronic microvascular injury. The “cytokine storm”, a systemic surge of IL-6, TNF-α, and IL-1β, precipitates widespread endotheliitis characterized by direct viral-mediated endothelial inflammation. This activates the complement cascade and extrinsic coagulation pathway, establishing a pro-thrombotic milieu within intramyocardial arterioles. The resulting microthrombosis and capillary leakage severely impair coronary microcirculatory reserve, producing myocardial ischemia even without obstructive CAD [122]. Unlike traditional atherosclerosis, virus-induced CMD involves rapid, systemic injury to the endothelial glycocalyx [33], blunting hyperemic responses and predisposing patients to long-term cardiovascular sequelae. From a clinical perspective, these mechanisms provide a plausible pathological basis for both INOCA and MINOCA. Persistent endothelial inflammation after viral infection may also underlie chronic exertional angina or HF symptoms in a subset of patients, thereby linking post-viral CMD to the broader syndrome of HFpEF [123].

### 2.9 Dyslipidemia: Lipotoxicity and Sterile Inflammation

Beyond its well-established role in epicardial atherogenesis, dyslipidemia creates a lipotoxic microenvironment that directly impairs the microcirculation. Oxidized low-density lipoprotein (oxLDL) serves as a sterile danger signal, binding scavenger receptor CD36 on microvascular ECs. This interaction triggers NLRP3 inflammasome assembly and pro-inflammatory chemokine release [96]. Specifically, the oxLDL-CD36 axis amplifies oxidative stress and diminishes NO bioavailability, impairing endothelium-dependent vasodilation. In patients with severe dyslipidemia, this persistent inflammatory insult culminates in microvascular wall thickening and a significant reduction in capillary density, establishing CMD as a key mechanism of lipid-mediated myocardial damage and contributing to myocardial dysfunction and HFpEF.

## 3. Diagnostic Evaluation of CMD: Correlation between Inflammatory and Functional Indicators

The clinical utility of inflammatory mechanisms in CMD depends on their robust correlation with functional diagnostic parameters. Integrating molecular pathways with physiological assessments, using both invasive and non-invasive modalities, is essential for quantifying microvascular impairment and its underlying inflammatory burden.

### 3.1 Invasive Functional Indices

CFR and the index of microcirculatory resistance (IMR) remain the gold-standard invasive metrics for CMD. Clinically, a CFR <2.0 or an IMR >25 indicates significant microvascular derangement [124]. In the setting of chronic inflammation, systemic inflammatory stress directly impairs microcirculatory conductance, as demonstrated by a significant inverse correlation between circulating hs-CRP or IL-6 levels and CFR [125].

### 3.2 Non-Invasive Imaging

Positron Emission Tomography (PET) enables absolute quantification of myocardial blood flow (MBF) and myocardial flow reserve (MFR), allowing for the detection of subclinical microvascular compromise before overt symptom onset. Although PET offers high diagnostic accuracy in INOCA, MINOCA, and HFpEF cohorts, its clinical use is limited by radiation exposure, costs, and restricted availability [126]. Cardiovascular Magnetic Resonance (CMR) has emerged as a comprehensive alternative, providing structural and functional characterization. The CMR-derived MPRI serves as a validated surrogate for invasive CFR, facilitating differentiation between epicardial and microvascular disease [125,127]. Additionally, CMR-based T1 and T2 mapping sensitively detect diffuse myocardial fibrosis and edema, which represent the key histological hallmarks of inflammation-mediated remodeling.

### 3.3 Inflammatory Biomarkers

Circulating biomarkers, including hs-CRP, cardiac troponin T (cTnT), IL-6, and tumor necrosis factor-alpha (TNF-α), reflect the severity of CMD and serve as independent predictors of adverse outcomes. When combined with functional indices, these biochemical markers significantly enhance diagnostic precision and enable more granular risk stratification in patients with suspected microvascular disease [125].

## 4. Therapeutic Interventions: Targeting the Inflammatory Axis in CMD

The pathogenic architecture of CMD encompasses structural microvascular remodeling, functional vasomotor impairment, and excessive oxidative stress, all driven by converging systemic and localized pro-inflammatory mediators [7,8]. Given the intrinsic link between these pathological features and chronic inflammatory signaling, attenuating systemic inflammation represents a viable strategy for restoring microvascular homeostasis. The current pharmacological armamentarium spans conventional cyclooxygenase (COX) inhibitors and colchicine to cytokine-targeting biologics and pleiotropic metabolic modulators such as SGLT2i [128].

### 4.1 COX Inhibitors: The Balance of Eicosanoid Signaling

Nonsteroidal anti-inflammatory drugs (NSAIDs) exert therapeutic efficacy through competitive inhibition of COX isoforms, attenuating pro-inflammatory eicosanoid biosynthesis [129].

Non-selective NSAIDs, including aspirin, remain cornerstones of antiplatelet therapy and have been proven effective for secondary prevention of epicardial atherosclerotic diseases; however, their systemic anti-inflammation use is limited by COX-1-mediated adverse effects, particularly gastrointestinal toxicity [124]. Paradoxically, high-dose salicylates may worsen endothelial dysfunction by reducing NO bioavailability [130]. Selective COX-2 inhibitors, such as celecoxib, offer a more targeted approach to localized inflammatory sites, potentially mitigating systemic adverse events while preserving potent anti-inflammatory activity within the microcirculatory environment.

### 4.2 Colchicine: A Pleiotropic Immunomodulator

Colchicine, a tricyclic alkaloid, disrupts multiple inflammatory nodes, primarily through the inhibition of the NLRP3 inflammasome [95,131]. By binding β-tubulin and arresting microtubule polymerization, colchicine impedes spatial assembly of the NLRP3 complex, thereby curtailing the maturation of IL-1β and IL-18 [132,133].

Beyond inflammasome suppression, colchicine modulates leukocyte-endothelial interactions by downregulating selectins and inhibiting neutrophil chemotaxis [134]. Clinical data, notably from the COLCOT trial, suggest that microtubule interference effectively reduces microvascular inflammatory burden and ischemic event incidence [135].

However, a comprehensive review by D’Amario et al. [136] critically appraised the “good, the bad and the ugly” of colchicine in ischemic heart disease, noting that while trials such as COLCOT and LoDoCo2 demonstrated reductions in major adverse cardiovascular events (MACE), these benefits were driven primarily by prevention of epicardial plaque-related events rather than direct microvascular protection. The distinction between epicardial and microvascular effects is clinically important. In acute myocardial infarction, the CLEAR SYNERGY trial found that colchicine initiated within 72 hours and continued for a median of 3 years did not reduce the composite primary outcome (cardiovascular death, recurrent MI, stroke, or unplanned revascularization; HR 0.99, 95% CI 0.85–1.16, *p* = 0.93), with diarrhea as the predominant adverse effect [137]. Conversely, in stable settings, the COPE-PCI substudy showed that pre-procedural colchicine significantly improved post-PCI CFR compared with placebo (3.25 vs 2.00, *p* = 0.03) [138]. These discordant findings suggest that colchicine’s microvascular benefits may be confined to chronic low-grade inflammatory states, whereas acute ischemia-reperfusion injury may be less amenable to delayed inflammasome inhibition. The ongoing COL-Micro-HF trial (NCT06217120) is evaluating 6-month colchicine treatment in 60 patients with HF and LV ejection fraction >40%, aiming to establish its potential role in improving CMD as assessed by CFR [139].

### 4.3 Precision Cytokine-Targeting Biologics

Inflammation is central to atherosclerosis, pericarditis, acute myocardial infarction, and HF. The NLRP3 inflammasome drives the production of IL-1β, and IL-1β blockade has proven effective in these conditions, supporting targeted anti-inflammatory therapy as a potential strategy for the management of CMD [140]. Cytokine-targeting biologics enable precise intervention at key inflammatory hubs. TNF-α antagonists (e.g., adalimumab) and IL-1β inhibitors (e.g., canakinumab) have reduced MACE in high-risk populations [141,142]. In the CANTOS trial, canakinumab reduced recurrent cardiovascular events in post-MI patients with elevated hs-CRP, independent of lipid-lowering [142]. In the CMD context, the IL-6 receptor inhibitor tocilizumab has attenuated microvascular obstruction (MVO) in the acute phase, suggesting a potent vasoprotective effect [143]. However, MVO and CMD are pathophysiologically distinct, and whether these agents confer direct benefit on CMD remains unestablished. Moreover, the clinical trade-off between enhanced anti-inflammatory efficacy and increased infection risk necessitates careful patient selection.

### 4.4 SGLT2i: A Paradigm Shift in Immunometabolic Protection

Originally developed as glucose-lowering agents, SGLT2i have become a cornerstone of cardiovascular protection. SGLT2i exert systemic and localized anti-inflammatory effects by modulating the “immunometabolic axis” [144]. At the cellular level, they suppress NLRP3 inflammasome activation in ECs and macrophages, thereby preserving mitochondrial integrity and enhancing NO bioavailability [145,146]. Although mechanistic evidence supports microvascular improvement, major SGLT2i efficacy trials were not designed to evaluate CMD-specific endpoints. Accordingly, SGLT2i may be particularly effective in CMD patients with comorbid diabetes, HF, or chronic kidney disease, but their role as direct anti-inflammatory therapy targeting CMD requires further confirmation [147].

### 4.5 Natural Products and Lipid-Modulating Strategies

Phytomedicines, including tanshinones and curcumin, represent emerging multi-target candidates that attenuate inflammatory progression by activating AMP-activated protein kinase (AMPK) signaling and scavenging ROS [148,149,150,151,152,153,154]. Furthermore, lipid-modulating pharmacotherapy, particularly statins, provides non-canonical “pleiotropic” benefits, including endothelial stabilization and immunomodulation, that transcend simple cholesterol lowering [155]. Multiple studies confirm that statins improve CFR in microvascular angina and significantly prolong the time to >1-mm ST-segment depression during stress testing [156,157,158]. Strategic implementation of these agents addresses both metabolic derangement and vascular inflammation, offering a comprehensive approach to preserving microvascular integrity.

## 5. AI in CMD: Prediction and Prognosis

AI and machine learning (ML) have shown considerable promise in diagnosing, risk-stratifying, and prognostically evaluating CMD.

In patients with ST-segment elevation myocardial infarction (STEMI) undergoing primary percutaneous coronary intervention (PPCI), an explainable ML model incorporating angiography-derived microvascular resistance (AMR) and clinical features achieved an area under the curve (AUC) of 0.846 for predicting MVO in the validation set, with XGBoost yielding the best performance. SHAP analysis further identified multiple contributing factors, including microvascular resistance, systolic blood pressure, and pre-procedural Thrombolysis in Myocardial Infarction (TIMI) flow grade [159]. Another AI model using pre- and post-PCI electrocardiographic (ECG) features predicted CMR-confirmed MVO with an AUC of 0.83, specificity of 94%, and sensitivity of 60%, supporting its utility for rapid, noninvasive risk stratification [160]. Furthermore, a convolutional neural network (CNN)-based model further achieved 91.3% feasibility in computing CFR directly from coronary angiography, offering a wire-free, adenosine-free approach to microvascular assessment [161].

However, current AI applications remain predominantly diagnostic and prognostic, with limited studies predicting therapeutic response in CMD. Future multimodal AI models integrating imaging, electrocardiography, inflammatory biomarkers, and multi-omics data are expected to enable personalized prediction of treatment efficacy and advance precision management.

## 6. Conclusion and Future Perspectives

CMD has emerged as a pivotal convergent phenotype across the cardiovascular spectrum, with pathogenesis orchestrated by a chronic, low-grade inflammatory continuum. This review has delineated how diverse clinical entities, encompassing hemodynamic stressors, metabolic derangements, and systemic immune activation, converge to compromise coronary microvascular structure and function. Despite divergent primary etiologies, these disorders intersect at conserved pathogenic hubs that collectively drive microcirculatory failure. Central to this process is the NLRP3 inflammasome, which functions as a high-sensitivity molecular sensor, converting proteotoxic, metabolic, and microbial stressors into sustained release of IL-1β and IL-18, directly provoking endotheliitis and microthrombogenesis. This inflammatory state is amplified by oxidative-inflammatory synergy, wherein mechanical and metabolic stress elicit excessive ROS production, sustaining NF-κB transactivation, subsequently degrading the endothelial glycocalyx and compromising barrier integrity. These signaling cascades lead to the universal uncoupling of NO pathways, wherein blunted eNOS activity and accelerated NO scavenging abrogate the physiological “vasodilatory brake”, severely attenuating CFR. Ultimately, this sustained molecular derangement triggers a profound architectural maladaptation, characterized by vascular smooth muscle cell phenotypic switching and EndMT, culminating in irreversible microvascular rarefaction. Crucially, inflammation in CMD is not merely ancillary but a primary driver of a vicious feed-forward loop in which microvascular impairment and systemic inflammation mutually reinforce each other throughout the disease continuum. Identifying these shared inflammatory nodes provides a mechanistic rationale for cross-disease therapeutic strategies. Future research must prioritize the integration of multi-omics immunophenotyping and advanced functional imaging to define specific “inflammatory endotypes”. Such precision-based approaches will be essential to translate current mechanistic insights into personalized anti-inflammatory regimens, ultimately improving clinical management and long-term outcomes in patients with coronary microcirculatory failure.
